# Effect of Pulsed Field Ablation System and Post-Ablation Mapping on Atrial Fibrillation Recurrence

**DOI:** 10.3390/jcdd13060243

**Published:** 2026-06-02

**Authors:** Benjamin J. Behers, Christoph A. Stephenson-Moe, Sammy Shihadeh, Tonya S. King, Omar Hozayen, Joseph Hozayen, Maria Moreno, Karen M. Hamad, Antonio Moretta

**Affiliations:** 1 Florida State University Internal Medicine Residency at Sarasota Memorial Hospital, Sarasota, FL 34239, USA; cstephensonmoe@med.fsu.edu (C.A.S.-M.); omar.hozayen@med.fsu.edu (O.H.); yousef.hozayen@med.fsu.edu (J.H.); karen-hamad@smh.com (K.M.H.); 2 Department of Clinical Sciences, Florida State University College of Medicine, Tallahassee, FL 32304, USA; 3 Kolschowsky Research & Education Institute, Sarasota Memorial Health Care System, Sarasota, FL 34239, USA; tonya-king@smh.com (T.S.K.); maria-moreno@smh.com (M.M.); 4 Sarasota Memorial Hospital, Sarasota, FL 34239, USA; moretta.md@gmail.com

**Keywords:** pulsed field ablation, circular catheter, pentaspline catheter, atrial fibrillation

## Abstract

Atrial fibrillation (AF) is the most common arrhythmia worldwide and is associated with significant morbidity and mortality. Catheter ablation of AF has been shown to result in a significant reduction in AF burden and recurrence. Pulsed field ablation (PFA) is a new modality of catheter ablation that is noninferior to its thermal ablation counterparts, coupled with a more favorable safety profile. This study seeks to compare clinical outcomes between two PFA systems: PulseSelect™ (Medtronic, Minneapolis, MN, USA) (circular catheter) and FARAPULSE™ (Boston Scientific, Marlborough, MA, USA) (pentaspline catheter). Secondary aims are to evaluate the impacts of post-ablation mapping with a high-density mapping catheter (PAHDMC) and both procedure and fluoroscopy times on recurrence. Overall, across 895 patients with a median follow-up of 12.5 months, there was a recurrence rate of 39%. PFA system, PAHDMC, and procedure time all had no effect on recurrence. To our knowledge, this is the first study to compare recurrence rates between different PFA systems. Fluoroscopy time, however, was a significant predictor of recurrence. In the pentaspline catheter group, the odds of recurrence were 60% greater for every 15 min increase in fluoroscopy time. Future studies are needed to continue comparing outcomes amongst PFA systems and assess whether PAHDMC improves outcomes in PFAs.

## 1. Introduction

Atrial fibrillation (AF) is the most common arrhythmia worldwide, with an estimated prevalence of 52.55 million cases, and is associated with significant morbidity and mortality [1]. Catheter ablation of AF, an important rhythm control strategy, is the most performed cardiac ablation procedure worldwide [2]. Studies have shown catheter ablation results in a significant reduction in both AF burden and recurrence rates compared to pharmacologic therapy, resulting in improved quality of life with less symptoms and increased functional capacity [3]. Although no mortality benefit has been demonstrated for the catheter ablation of AF in patients without left ventricular dysfunction, ablation has been shown to reduce health care use and costs [3]. Catheter ablation is indicated in symptomatic, recurrent paroxysmal, or persistent AF refractory to antiarrhythmic drugs [4].

Pulsed field ablation (PFA) is a new modality of catheter ablation using rapid, high-voltage electrical pulses that cause electroporation and ultimately the necrosis of cardiac myocytes [5]. Due to its specificity for myocytes, PFA confers less side effects than its thermal ablation counterparts [5]. Furthermore, the ADVENT trial showed PFA to be noninferior to thermal ablation (radiofrequency and cryoballoon) in terms of procedure efficacy, recurrence, and safety data [5]. These findings led to the approval of two PFA systems in the United States and Europe: PulseSelect™ (Medtronic, Minneapolis, MN, USA) and FARAPULSE™ (Boston Scientific, Marlborough, MA, USA) [6]. PulseSelect™ is characterized by an over-the-wire circular catheter, while FARAPULSE™ is characterized by a pentaspline catheter [6].

Although PFA has yielded promising results overall, studies lack a comparison of different PFA systems and techniques. This study seeks to compare clinical outcomes between two PFA systems: PulseSelect™ (circular catheter) and FARAPULSE™ (pentaspline catheter). Additionally, there are two secondary aims: (1) to evaluate the impact of post-ablation mapping with a high-density mapping catheter (PAHDMC) on recurrence and (2) to evaluate the impact of procedure and fluoroscopy times on recurrence.

## 2. Materials and Methods

This retrospective cohort study was approved by our Institutional Review Board prior to commencement. Cases of PFA at our institution in 2024 were identified and a chart review with data extraction was performed. Patients who underwent prior AF ablations were excluded. Data extracted were patient demographics, type of PFA catheter used (circular vs. pentaspline), whether PAHDMC was performed, type of AF (paroxysmal vs. persistent), echocardiographic findings at the time of ablation, procedure and fluoroscopy times in minutes (min), and recurrence. Patient demographics of interest were age at time of ablation and gender. PAHDMC was characterized by the use of Boston Scientific’s IntellaMap Orion™ (Marlborough, MA, USA), Abbott’s Advisor HD Grid™ (Plymouth, MN, USA), or Johnson & Johnson’s OCTARAY™ (Irvine, CA, USA). Echocardiographic findings included the left atrial (LA) volume (mL/m^2^) as determined by the left atrial volume index on a 4-chamber echocardiogram, the left ventricular ejection fraction (LVEF), and the presence of severe mitral regurgitation (MR). Recurrence was defined by either repeat ablation, ongoing antiarrhythmic drug (AAD) use, cardioversion, or hospitalization/emergency department (ED) visits for AF after the one-month blanking period [7].

Demographic and baseline clinical characteristics were summarized using a median with an interquartile range (IQR) for continuous variables and frequencies for categorical variables. These characteristics were compared between PFA system groups using Kruskal–Wallis tests for continuous measures and Fisher’s Exact tests for categorical measures. Recurrence was the primary outcome, while the individual components of recurrence and procedure and fluoroscopy times were secondary outcomes. Groups were compared with respect to clinical outcomes using logistic regression for dichotomous outcomes and analysis of covariance for continuous outcomes, with adjustment for baseline factors that were significantly different between groups. Interaction terms were evaluated between the PFA groups and baseline factors. If necessary, nested effects were estimated within each model framework to aid in the proper interpretation of the results. Results were reported as adjusted odds ratios and adjusted means, with corresponding 95% confidence intervals (95% CI). Procedure time and fluoroscopy time were evaluated as predictors of recurrence, repeat ablation, and hospitalization/ED visits by including each time measure in each logistic regression model, with adjustment for PFA type. Sensitivity analyses were performed using propensity-matched analysis with matching for age, sex, PAHDMC, and AF type. Additionally, a sensitivity analysis was performed between initial and more recent cases for each operator. Significance was defined as *p* < 0.05. Statistical analyses were performed using SAS statistical analysis software version 9.4 (SAS Institute Inc., Cary, NC, USA).

## 3. Results

A total of 1242 PFAs were performed at our facility in 2024. There were 347 patients that had undergone previous AF ablations and were excluded, resulting in 895 patients included in this study. Of these 895 patients, 162 underwent ablations with the circular catheter and 733 with the pentaspline catheter (Table 1). Median age and gender were not significantly different between the two groups. However, patients undergoing ablation with the circular catheter had a significantly higher percentage of PAHDMC (69.1% vs. 44.2%; *p* < 0.001). Additionally, AF type was significantly different between groups (*p* < 0.001). This difference appears to be driven by a higher percentage of paroxysmal AF in the circular catheter group (67.3% vs. 55.0%). These factors were taken into account when comparing outcomes between groups. LA volume, LVEF, and severe MR were not significantly different between groups.

Overall, there was a 39% recurrence rate over a median follow-up time of 12.5 (IQR: 11.6, 13.8) months (Table 2). However, this varied amongst individual components of recurrence from 7.4% of patients requiring cardioversion to 31.2% with ongoing AAD use, as well as 10.2% undergoing repeat ablation. Recurrence rates amongst the pentaspline and circular catheter groups were 39.4% and 37.0%, respectively. The PFA system groups were not significantly different with respect to the odds of recurrence, the odds of repeat ablation, or the odds of hospitalization/ED visit for AF.

Overall median procedure and fluoroscopy times were 54.0 and 12.4 min, respectively (Table 3). There was significant interaction between PFA type and PAHDMC for the time outcomes (*p* < 0.001 for both), which means the PFA types need to be compared separately for those with and without PAHDMC for each outcome. The circular catheter group had a longer average procedure time than the pentaspline group in both those with PAHDMC (70.8 vs. 63.8; *p* = 0.019) and without PAHDMC (78.8 vs. 44.3; *p* < 0.001). Conversely, the circular catheter group had a shorter average fluoroscopy time than the pentaspline catheter group for those with PAHDMC (7.7 vs. 14.8; *p* < 0.001) and a longer average fluoroscopy time for those who did not have PAHDMC (14.3 vs. 12.1; *p* = 0.049). 

PAHDMC had no significant effect on the odds of recurrence, the odds of repeat ablation, or the odds of hospitalization/ED visit (Table 4). In the circular catheter group, PAHDMC had no significant effect on average procedure time, but was associated with a shorter average fluoroscopy time (7.7 vs. 14.3, *p* < 0.001). In the pentaspline catheter group, PAHDMC was associated with both a longer average procedure time (63.8 vs. 44.3, *p* < 0.001) and longer average fluoroscopy time (14.8 vs. 12.1, *p* < 0.001).

Procedure time was not a significant predictor of recurrence for either PFA type (Table S1). Fluoroscopy time was a significant predictor of recurrence (*p* = 0.014) and, while the interaction between fluoroscopy time and PFA type was not significant (*p* = 0.17), the relationship was different for the circular and pentaspline catheter groups. There was a significant positive relationship for the pentaspline group, but no significant relationship for the circular group. In the pentaspline catheter group, the odds of recurrence were 1.6 times or 60% greater for every 15 min increase in fluoroscopy time. However, neither procedure time nor fluoroscopy time were significant predictors of repeat ablation or hospitalization/ED visit.

We performed a propensity-matched analysis that included all 162 participants from the circular catheter group and 473 from the pentaspline group. Results of the propensity-matched analyses were nearly identical to the covariate-adjusted analysis for PFA type, PAHDMC, procedure time, and fluoroscopy time on recurrence (Tables S2–S4). This study included data from eight operators. However, there were only three operators with at least 12 cases using the circular catheter. Comparison of the initial and most recent cases with the circular catheter showed a significant reduction in both median procedure time (95.0 vs. 68.0) and fluoroscopy time (20.6 vs. 10.0), as well as nonsignificant increases in recurrence, repeat ablation, and hospitalization/ED visit (Table S5). Alternatively, there were seven operators with at least 20 cases using the pentaspline catheter. Comparison of the initial and most recent 10 cases with the pentaspline catheter showed significant reductions in median procedure time (67.5 vs. 55.0), median fluoroscopy time (18.6 vs. 9.7), and repeat ablation (15.7% vs. 4.3%), although there was no significant reduction in overall recurrence or hospitalization/ED visit (Table S6).

## 4. Discussion

Across 895 patients over a median follow-up time of 12.5 months, overall recurrence was 39%. Recurrence was primarily driven by the ongoing use of AAD at a rate of 31.2%. PFA type (circular vs. pentaspline catheter), PAHDMC, and procedure time had no significant effect on overall recurrence, nor the odds of repeat ablation or hospitalization/ED visit for AF. However, fluoroscopy time was a significant predictor of recurrence, with the odds of recurrence in the pentaspline group being 60% greater for every 15 min increase in fluoroscopy time. Procedure time was longer in the circular catheter group, regardless of the use of PAHDMC. Alternatively, fluoroscopy time between PFA groups was dependent upon PAHDMC. PAHDMC led to shorter average fluoroscopy times in the circular catheter group, while a lack of PAHDMC led to shorter average fluoroscopy times in the pentaspline catheter group. To our knowledge, this is the first study to investigate recurrence rates between different PFA systems.

Although studies evaluating recurrence between PFA systems are lacking, numerous studies investigating acute procedural outcomes exist. An international, multicenter study of 402 patients found that the pentaspline catheter was associated with a significantly shorter procedure time than the circular catheter (36.0 vs. 49.0 min), while there was no significant difference in fluoroscopy time [6]. Another study of 120 patients found that the pentaspline catheter was associated with both a significantly shorter procedure time (57 vs. 66 min) and fluoroscopy time (11 vs. 14 min) than the circular catheter [8]. A third study may help explain the variation between PFA systems, as it found that the circular catheter required more energy applications to achieve pulmonary vein isolation than the pentaspline catheter (35.0 vs. 32.0) [9]. Notably, acute procedural success, defined as the electrical isolation of all ablation targets, was achieved by both PFA systems in all three of these studies [6,8,9]. These findings are similar to the results obtained in our study, suggesting both systems are equally effective in performing AF ablation, although the circular catheter is typically associated with longer procedure times.

These observed differences between the pentaspline and circular catheters can likely be attributed to their differing structures. The circular catheter consists of nine electrodes that are 25 mm in diameter, while the pentaspline catheter consists of 20 electrodes that are 31 or 35 mm in diameter [6]. While the circular catheter takes the shape of either a forward-tilted lasso or spiral shape, the pentaspline catheter can undergo various configurations, including linear, a spherical basket, or a fully deployed flat flower [6,8]. Furthermore, pulse delivery variations exist between the two systems, with the circular catheter delivering four biphasic, bipolar pulses at 1.5 kV over 100–200 ms per pulse, compared to pentaspline catheters delivering five biphasic, bipolar pulses at 2 kV over 2.5 s [6,8]. Additionally, variations exist in energy application procedures between the two systems. The pentaspline catheter typically delivers four applications in both the basket and flower configurations in two overlapping positions, while the circular catheter typically delivers both four ostial and four antral applications in 90-degree rotated positions [6,8].

Given the observed differences in procedure and fluoroscopy times, further understanding of the clinical implications of these is important. PFA is generally performed under either deep sedation or general anesthesia, both of which are associated with sedation-related complications, such as airway obstruction and hypoventilation, hypoxia and hypercapnia, and hemodynamic instability [10]. Additionally, fluoroscopy is associated with complications due to increased radiation exposure to both the patient and provider, the most notable of which is carcinogenesis [11]. Recognition of the deleterious effects associated with increased procedure and fluoroscopy times highlights the importance of taking these aspects into consideration when comparing PFA systems.

Aside from the PFA system, we also observed no effect on recurrence with PAHDMC. Post-ablation mapping with a 3-dimensional electroanatomic map was utilized to ensure pulmonary vein isolation [12]. Previous studies have found PAHDMC to be associated with shorter procedure and fluoroscopy times, enhanced safety, and lower rates of arrhythmia recurrence [13]. However, studies focused solely on PFA are scarce and yield mixed results. One study of 1804 participants found that post-ablation mapping was associated with increased procedure and fluoroscopy times, while providing no significant reduction in recurrence rates over a median follow-up of 395 days [14]. Another study of 248 participants found that post-ablation mapping resulted in a significant reduction in fluoroscopy time, although there was no significant difference in procedure time [15]. Interestingly, in our study, results differed between PFA systems. PAHDMC was associated with both shorter average procedure and fluoroscopy times in the circular catheter group, compared with both longer average procedure and fluoroscopy times in the pentaspline group. Future studies are needed into whether PAHDMC offers benefits on clinical and procedural outcomes in PFA, as well as whether differences in its use are observed between PFA systems.

As we have alluded to, our overall recurrence rate of 39% was greatly influenced by our inclusion of ongoing AAD use as a proxy for recurrence. In contrast, overall rates of cardioversion and repeat ablation, which represent more definitive evidence of recurrence, were only 7.4% and 10.2%, respectively. Despite this, our overall recurrence rate is similar to other published studies. Trial data has shown PFA to have recurrence rates ranging from 21.5% to 44.9%, while observational studies have yielded recurrence rates of 18.2% and 21.9% [16]. Risk factors for AF recurrence have been studied extensively. Type of atrial fibrillation seems to be the strongest predictor, with persistent being associated with higher recurrence rates than paroxysmal [16]. Additionally, atrial enlargement and procedure time both have a positive association with recurrence [16]. However, to our knowledge, our study is the first to link longer fluoroscopy time to increased risk of recurrence. While increased fluoroscopy time could reflect greater procedural complexity, challenging anatomy, or operator-related factors, the lack of corresponding increased procedural time is interesting. Given these confounding factors, our relationship between fluoroscopy time and recurrence should be interpreted with caution. Future studies should continue to assess whether fluoroscopy time can be independently predictive of recurrence. Furthermore, future studies should continue to assess long-term recurrence rates following PFA, as well as factors associated with both better and worse outcomes.

Our study is not without limitations, the greatest of which is its retrospective cohort design, which is inherently prone to bias. This study design forced us to rely on available data within our system, likely leading to inaccuracies with our recurrence data. Future studies should utilize continuous cardiac monitoring for the most accurate assessment of recurrence. Since our included population did not have continuous monitoring, we included ongoing AAD use as part of our recurrence outcome, likely vastly overestimating true recurrence. Another limitation is that we did not control for different ablation operators, which may have differing skill levels, as well as different preferences in terms of PFA system and use of PAHDMC. Our sensitivity analysis comparing initial cases to the most recent cases for each catheter showed improvements in both procedure and fluoroscopy time, highlighting the operator learning curve, which may have influenced our results. Additionally, significant variations in sample size exist between PFA system groups, which may have affected our overall results. Despite statistical adjustment and the use of a propensity-matched sensitivity analysis, the possibility of residual confounding remains substantial, and our results thus must be interpreted with caution. Furthermore, while our median follow-up time was 12.5 months, this represents a short period of time in terms of AF recurrence. Future studies should pursue longer follow-up periods to better reflect clinically significant recurrence rates. Finally, the fact that our study was single-centered and did not control for variations among participants limits the generalizability of our findings.

## 5. Conclusions

Our study suggests that both PFA system (circular vs. pentaspline catheters) and use of PAHDMC have no effect on arrhythmia recurrence following catheter ablation for AF. However, fluoroscopy time was a significant predictor of recurrence. Procedure time was longer in the circular catheter group regardless of the use of PAHDMC, while fluoroscopy times between PFA systems was dependent on the use of PAHDMC. Future studies are needed to continue comparing clinical and procedure outcomes amongst PFA systems, as well as to assess whether PAHDMC improves outcomes in PFA.

## Figures and Tables

**Table 1:** Demographics and baseline clinical factors in each group.

Characteristic			PFA Type		
		Overall (N = 895)	Circular (N = 162)	Pentaspline (N = 733)	*p*-Value
Age		73.0 (67.0, 78.0)	73.0 (67.0, 77.0)	73.0 (67.0, 79.0)	0.40 ^1^
Sex	Male	526 (58.8%)	94 (58.0%)	432 (58.9%)	0.86 ^2^
	Female	369 (41.2%)	68 (42.0%)	301 (41.1%)	
PAHDMC	Yes	436 (48.7%)	112 (69.1%)	324 (44.2%)	<0.001 ^2^
	No	459 (51.3%)	50 (30.9%)	409 (55.8%)	
AF Type	Paroxysmal	512 (57.2%)	109 (67.3%)	403 (55.0%)	<0.001 ^2^
	Persistent	273 (30.5%)	48 (29.6%)	225 (30.7%)	
	NR	110 (12.3%)	5 (3.1%)	105 (14.3%)	
LA Vol (mL/m^2^)		41.1 (28.8, 62.5)	40.2 (25.9, 57.3)	41.5 (29.3, 63.7)	0.35 ^1^
EF		55.0 (50.0, 60.0)	55.0 (50.0, 60.0)	55.0 (50.0, 60.0)	0.17 ^1^
Severe MR	Yes	16 (1.8%)	4 (2.5%)	12 (1.6%)	0.81 ^2^
	No	654 (73.1%)	120 (74.1%)	534 (72.9%)	
	NR	225 (25.1%)	38 (23.5%)	187 (25.5%)	
^1^ Kruskal–Wallis *p*-value; ^2^ Fisher Exact *p*-value Data is reported in n (%) or median (IQR).					

PFA Type: Pulsed field ablation catheter used; PAHDMC: Post-ablation mapping with a high-density mapping catheter; AF type: Atrial fibrillation type (paroxysmal vs. persistent); NR: Not reported; LA Vol: Left atrial volume as determined by left atrial volume index on 4-chamber echocardiogram; EF: Ejection fraction on echocardiogram; Severe MR: Presence of severe mitral regurgitation on echocardiogram.

**Table 2:** Effect of PFA type on recurrence.

		PFA Type			
Outcome	Overall (N = 895)	Circular (N = 162)	Pentaspline (N = 733)	Circular vs. Pentaspline	*p*-Value
Recurrence	349 (39.0%)	60 (37.0%)	289 (39.4%)	0.9 (0.65, 1.35)	0.73
Repeat Ablation	91 (10.2%)	16 (9.9%)	75 (10.2%)	1.1 (0.60, 1.95)	0.80
Hosp/ED Visit	98 (10.9%)	15 (9.3%)	83 (11.3%)	0.9 (0.47, 1.55)	0.60
Cardioversion	66 (7.4%)	9 (5.6%)	57 (7.8%)		
Data is presented as n (%) and OR (95% CI), adjusted for PAHDMC and AF type as covariates in logistic and linear regression models.					

PFA Type: Pulsed field ablation catheter used; Hosp/ED Visit: Patients who had a hospitalization or emergency department visit for atrial fibrillation; PAHDMC: Post-ablation mapping with a high-density mapping catheter; AF type: Atrial fibrillation type (paroxysmal vs. persistent).

**Table 3:** Effect of PFA type on procedure and fluoroscopy times.

			PFA Type		
	Overall	PAHDMC	Circular	Pentaspline	*p*-Value
Procedure Time (min)	54.0 (37.0, 73.0) *	Yes	70.8 (65.45, 76.09)	63.8 (60.44, 67.16)	0.019
		No	78.8 (71.01, 86.57)	44.3 (41.55, 47.08)	<0.001
Fluoroscopy Time (min)	12.4 (7.7, 17.8) *	Yes	7.7 (6.29, 9.19)	14.8 (13.92, 15.76)	<0.001
		No	14.3 (12.20, 16.46)	12.1 (11.35, 12.87)	0.049
Data is presented as mean (95% CI), adjusted for PAHDMC and AF type as covariates in logistic and linear regression models. * Data is presented as median (IQR).					

PFA Type: Pulsed field ablation catheter used; PAHDMC: Post-ablation mapping with a high-density mapping catheter; min: minutes; CI: Confidence interval; AF type: Atrial fibrillation type (paroxysmal vs. persistent); IQR: Interquartile range.

**Table 4:** Effect of PAHDMC on clinical outcomes.

	PAHDMC		Odds Ratio (95% CI)	
Outcome	Yes (N = 436)	No (N = 459)	Yes vs. No	*p*-Value
Recurrence	166 (38.1%)	183 (39.9%)	0.9 (0.66, 1.16)	0.34
Repeat Ablation	40 (9.2%)	51 (11.1%)	0.7 (0.47, 1.17)	0.20
Hosp/ED Visit	46 (10.6%)	52 (11.3%)	0.8 (0.54, 1.31)	0.45
	PFA Type	PAHDMC	No PAHDMC	*p*-Value
Procedure Time (min) *	Circular	70.8 (65.48, 76.09)	78.8 (71.01, 86.57)	0.08
	Pentaspline	63.8 (60.44, 67.16)	44.3 (41.55, 47.08)	<0.001
Fluoroscopy Time (min) *	Circular	7.7 (6.29, 9.19)	14.3 (12.20, 16.46)	<0.001
	Pentaspline	14.8 (13.92, 15.76)	12.1 (11.35, 12.87)	<0.001
Data is presented as n (%) and OR (95% CI), adjusted for PFA type and AF type as covariates in logistic and linear regression models. * Data is presented as mean (95% CI), adjusted for PFA type and AF type as covariates in logistic and linear regression models.				

PAHDMC: Post-ablation mapping with a high-density mapping catheter; CI: Confidence interval; Hosp/ED Visit: Patients who had a hospitalization or emergency department visit for atrial fibrillation; PFA Type: Pulsed field ablation catheter used; min: minutes; AF type: Atrial fibrillation type (paroxysmal vs. persistent).

## Data Availability

Data is available from the first author upon reasonable request.

## Supplementary Materials

The following supporting information can be downloaded at: https://www.mdpi.com/article/10.3390/jcdd13060243/s1, Table S1: Effect of procedure and fluoroscopy time on recurrence; Table S2: Propensity-matched analysis for PFA type; Table S3: Propensity-matched analysis for PAHDMC; Table S4: Propensity-matched analysis for the effect of procedure and fluoroscopy time on recurrence; Table S5: Comparison of initial and most recent cases using the circular catheter; Table S6: Comparison of initial and most recent cases using the pentaspline catheter.
