# Supplementary material for: Effect of Pulsed Field Ablation System and Post-Ablation Mapping on Atrial Fibrillation Recurrence
Source: J Cardiovasc Dev Dis. 2026 Jun 2;13(6):243. doi: 10.3390/jcdd13060243 (PMC13301595; doi:10.3390/jcdd13060243)
Supplement: Supplementary file 1 [file jcdd-13-00243-s001.zip › jcdd-4287602-supplementary.pdf]

Table S1: Effect of procedure and fluoroscopy time on recurrence.

Outcome	Predictor	Main Effect P-value	P-value for interaction between predictor and PFA type	Regression coefficient and p-value for association of predictor vs. outcome	
				Circular Catheter	Pentaspline Catheter
Recurrence	Procedure Time	0.49	0.95		
	Fluoroscopy Time	0.014	0.17	B = 0.005 (*p* = 0.72)	B = 0.030 (*p* = 0.006)
Repeat Ablation	Procedure Time	0.81	0.80		
	Fluoroscopy Time	0.51	0.76		
Hosp/ED Visit	Procedure Time	0.30	0.27		
	Fluoroscopy Time	0.47	0.23		

PFA Type: Pulsed field ablation catheter used; Hosp/ED Visit: Patients who had a hospitalization or emergency department visit for atrial fibrillation

Table S2: Propensity-matched analysis for PFA type.

Outcome	Circular vs. Pentaspline		P-value	
Recurrence	0.9 (0.64, 1.35)*		0.70	
Repeat Ablation	1.1 (0.58, 1.93)*		0.86	
Hosp/ED Visit	0.9 (0.46, 1.57)*		0.61	
	PAHDMC	Circular	Pentaspline	P-value
Procedure Time	Yes	71.7 (65.19, 78.27)**	64.9 (60.02, 69.74)**	0.030
	No	79.9 (70.75, 88.97)**	44.9 (38.51, 51.27)**	<0.001
Fluoroscopy Time	Yes	8.0 (6.17, 9.88)**	15.1 (13.76, 16.52)**	<0.001
	No	14.7 (12.07, 17.24)**	12.3 (10.44, 14.06)**	0.072

Data is propensity-matched analysis using age, sex, PAHDMC, and AF type.
*Data is adjusted OR (95% CI)
**Data is adjusted mean (95% CI)

Hosp/ED Visit: Patients who had a hospitalization or emergency department visit for atrial fibrillation; PAHDMC: Post-ablation mapping with a high-density mapping catheter; AF type: Atrial fibrillation type (paroxysmal vs. persistent); OR: Odds ratio; CI: Confidence interval

Table S3: Propensity-matched analysis for PAHDMC.

Outcome	PAHDMC Yes vs. No		P-value	
Recurrence	0.8 (0.59, 1.19)*		0.32	
Repeat Ablation	0.8 (0.44, 1.34)*		0.34	
Hosp/ED Visit	0.9 (0.50, 1.51)*		0.61	
	PFA Type	PAHDMC	No PAHDMC	P-value
Procedure Time (min)**	Circular	71.7 (65.19, 78.27)	79.9 (70.75, 88.97)	0.097
	Pentaspline	64.9 (60.02, 69.74)	44.9 (38.51, 51.27)	<0.001
Fluoroscopy Time (min)**	Circular	8.0 (6.17, 9.88)	14.7 (12.07, 17.24)	<0.001
	Pentaspline	15.1 (13.76, 16.52)	12.3 (10.44, 14.06)	<0.001
Data is propensity-matched analysis using age, sex, PAHDMC, and AF type.<br>*Data is adjusted OR (95% CI)<br>**Data is adjusted mean (95% CI)				

PAHDMC: Post-ablation mapping with a high-density mapping catheter; Hosp/ED Visit: Patients who had a hospitalization or emergency department visit for atrial fibrillation; PFA Type: Pulsed field ablation catheter used; AF type: Atrial fibrillation type (paroxysmal vs. persistent); OR: Odds ratio; CI: Confidence interval

Table S4: Propensity-matched analysis for the effect of procedure and fluoroscopy time on recurrence.

Outcome	Predictor	Main Effect P-value	P-value for interaction between predictor and PFA type	Regression coefficient and p-value for association of predictor vs. outcome	
				Circular Catheter	Pentaspline Catheter
Recurrence	Procedure Time	0.92	0.86		
	Fluoroscopy Time	0.053	0.23	B = 0.005 (*p* = 0.72)	B = 0.029 (*p* = 0.025)
Repeat Ablation	Procedure Time	0.92	0.75		
	Fluoroscopy Time	0.40	0.59		
Hosp/ED Visit	Procedure Time	0.61	0.32		

	Fluoroscopy Time	0.78	0.31		

PFA Type: Pulsed field ablation catheter used; Hosp/ED Visit: Patients who had a hospitalization or emergency department visit for atrial fibrillation

Table S5: Comparison of initial and most recent cases using the circular catheter.

Outcome	Sample Size	Initial	Most Recent	P-value
Procedure Time	26	95.0 (77.0, 108.0)	68.0 (61.0, 110.0)	0.019
Fluoroscopy Time	26	20.6 (7.3, 27.0)	10.0 (0.2, 15.3)	<0.001
Recurrence	26	7 (26.9%)	13 (50.0%)	0.11
Repeat Ablation	26	1 (3.8%)	6 (23.1%)	0.059
Hosp/ED Visit	26	0 (0%)	4 (15.4%)	-
Data is mean (95% CI) or n (%).				

Hosp/ED Visit: Patients who had a hospitalization or emergency department visit for atrial fibrillation; CI: Confidence interval

Table S6: Comparison of initial and most recent cases using the pentaspline catheter.

Outcome	Sample Size	Initial	Most Recent	P-value
Procedure Time	70	67.5 (56.0, 94.0)	55.0 (32.0, 76.0)	<0.001
Fluoroscopy Time	70	18.6 (14.0, 25.2)	9.7 (6.8, 13.0)	<0.001
Recurrence	70	31 (44.3%)	24 (34.3%)	0.26
Repeat Ablation	70	11 (15.7%)	3 (4.3%)	0.033
Hosp/ED Visit	70	12 (17.1%)	6 (8.6%)	0.16
Data is mean (95% CI) or n (%).				

Hosp/ED Visit: Patients who had a hospitalization or emergency department visit for atrial fibrillation; CI: Confidence interval
